# A country-level comparison of access to quality surgical and non-surgical healthcare from 1990-2016

**DOI:** 10.1371/journal.pone.0241669

**Published:** 2020-11-03

**Authors:** Taylor Wurdeman, Gopal Menon, John G. Meara, Blake C. Alkire

**Affiliations:** 1 Program in Global Surgery and Social Change, Harvard Medical School, Boston, MA, United States of America; 2 Miller School of Medicine, University of Miami, Miami, FL, United States of America; 3 Columbia University Medical Center Department of Surgery, New York, NY, United States of America; 4 Department of Plastic and Oral Surgery, Boston Children’s Hospital, Boston, MA, United States of America; 5 Department of Otolaryngology, Massachusetts Eye and Ear Institute, Boston, MA, United States of America; Duke Global Health Institute, UNITED STATES

## Abstract

**Background:**

The Healthcare Access and Quality (HAQ) index, developed by the Institute for Health Metrics and Evaluation, uses estimates of amenable mortality to quantify health system performance over time. While much is known about general health system performance globally, few studies have portrayed the performance of surgical systems. In order to quantify access to quality surgical care, evaluate changes over time, and link these changes to health care investments, surgical and non-surgical Health Access and Quality sub-indices were developed.

**Design:**

We categorized 32 amenable mortality causes as either surgical or non-surgical conditions. Using principal components analysis and scaled amenable mortality rates, we constructed a surgical and non-surgical Health Access and Quality sub-index. Using these sub-indices, relative improvement over time was compared. An expenditure model with country fixed effects was built to explore drivers of differences in relative improvement of sub-indices.

**Results:**

Compared to low-income countries, high-income countries have been 2.77 times more effective at improving surgical care (p < .05). Government expenditure on healthcare has a larger effect on improving surgical Health Access and Quality (p < 0.05) while development assistance for health has a larger effect on improving non-surgical Health Access and Quality (p < 0.05).

**Conclusions and relevance:**

Global health investment must prioritize strengthening health systems as opposed to the historically favored vertical programming. In order to achieve health equity in low-income countries, more focus should be placed on domestic financing of surgical systems. Health Access and Quality sub-indices can be used by countries to identify targets, monitor progress, and evaluate interventions aimed at improving access to quality surgical healthcare.

## Introduction

Health outcomes have markedly improved across the globe over the past 30 years. Longer life expectancy and significant reductions in maternal and infant mortality have been achieved in both high-income countries (HICs) and low- and middle-income countries (LMICs) alike [1–3]. Despite these gains, there are marked disparities in health outcomes across geographic regions and income groups [4–7]. The global health community has traditionally focused on disease-specific programs addressing communicable diseases such as tuberculosis, malaria, and HIV, whereas just 1.5% of development aid is directed to NCD care [8]. While programs focused on communicable diseases have demonstrated mixed success, they obtain a disproportionate amount of funding and are less likely to increase the capacity of health systems as they confront the epidemiologic transition. Health conditions that require a robust health system with appropriate infrastructure and specialist care, such as surgical conditions, cancer, trauma, and novel disease processes are less amenable to this vertical approach.

Historically overlooked as part of the global health investment strategy, conditions amenable to surgical, obstetric, and anesthesia care have more recently come to the fore as critical targets for capacity building and strengthening. The Lancet Commission on Global Surgery (LCoGS) defined several weaknesses in the present global surgical ecosystem, including the lack of access to surgical care, impoverishing expenditure associated with surgery, and the relative lack of specialist surgical, obstetric, and anesthesia providers in most LMICs [9]. Much of the gap in mortality in LMICs is related to surgical conditions, with 28–32% of the global burden of disease attributable to surgical conditions [10]. Without significant health system strengthening incorporating surgery, obstetrics, and anesthesia, attaining the Sustainable Development Goal’s targets related to maternal mortality, non-communicable diseases, and injuries is unlikely [11]. Surgery is cross-cutting, uniting diverse medical disciplines through its utilization as a treatment for nearly all major causes of disease and injury. As a result, robust surgical systems improve the ability of a health system to address the disease burden of all categories of disease.

In addition to calls to increase healthcare capacity and the range of services provided, there has been increasing recognition of the importance and impact of the quality of care delivered by health systems [12–14]. LMICs and LICs suffer disproportionately due to lower quality healthcare, with nearly eight million people dying each year due to low-quality healthcare systems and up to 2.6% loss of potential GDP in LICs due to these deaths [12]. The Institute for Health Metrics and Evaluation (IHME) has proposed a metric to quantify country-level estimates of access and quality over time termed the Healthcare Access and Quality (HAQ) index [15]. Derived from country-level mortality estimates of 32 diseases known to be amenable to quality healthcare systems, the HAQ index suggests a significant improvement in access to quality healthcare in all regions and income groups between 1990–2016. Similar to the uneven progress noted above in health outcomes, relative changes among disease-specific estimates, which comprise the HAQ index, vary significantly by disease and country. Given the unmet surgical need recently defined, there may be differences in relative progress between surgical and non-surgical conditions.

A renewed focus on health system strengthening in multinational health organizations and the recognized need to address surgical capacity has led to a need to understand how to measure access and quality for surgical conditions. The known lack of access to surgical care and the lack of attention to building high-quality health systems suggests that access to quality surgical care may be lacking in LMICs, especially when compared to non-surgical conditions. This study proposes a new method of measuring the gap in the rate of improvement between surgical and non-surgical access and quality by constructing country and year specific surgical HAQ and non-surgical HAQ sub-indices. With these condition-specific sub-indices, the gap in the improvement of surgical HAQ and non-surgical HAQ within a country and region is assessed over time. Finally, a panel data regression is performed to understand specific factors that contribute to the gap in improvement of surgical and non-surgical HAQ. We hypothesize that surgical HAQ has improved less rapidly than non-surgical HAQ for low-income countries over time.

## Methods

### Data sources

Data sources for this study included estimates from the Institute for Health Metrics and Evaluation (IHME) global burden of disease project, the Varieties of Democracy (VDEM) project and the World Bank. These sources provide country- and year-specific data, which we then combine to create a panel dataset. The IHME provides two sets of data: the Health Access and Quality (HAQ) index and the Global Health Spending database [16]. The HAQ dataset provides estimates of individual scaled cause values, which are based on relative differences in mortality among countries, as well as the composite HAQ score. It is important to note that Health Access and Quality is a proxy measure for access and quality, as the index relies on cause-specific mortality and cannot be disaggregated. The Global Health Spending dataset provides per capita development assistance and government expenditure on health. Data from the World Bank included urban population (% of total). Additionally, surgical indicators from the World Bank (Number of Surgical procedures per 100,000; Specialist Surgical Workforce per 100,000) were used for model validation. Data from the VDEM database included GDP per capita.

### Approach

This study involved constructing surgery and non-surgery specific HAQ sub-indices using principal component analysis (PCA), which mirrors the methodology in the 2017 HAQ study. Conditions amenable to personal healthcare, which were drawn from the Nolte and McKee list used in the original HAQ calculation, were included in the surgical HAQ index if they were a surgical condition, which is defined as a condition that would benefit from the involvement of a surgeon, obstetrician, and/or anesthesiologist. All other conditions were included in the non-surgical HAQ sub-index. Table 1 demonstrates the causes included in each sub-index. HAQ sub-indices were able to be estimated for 195 countries for the years 1990, 1995, 2000, 2005, 2010, and 2016. Following the construction of these specific HAQ sub-indices, gaps in improvement were assessed at the regional (GBD Super Region), income group (World Bank Income Group), and country levels. Panel data regression models were assessed to better understand the factors leading to gaps in surgical and non-surgical HAQ improvement. More information on the methods used for calculation of the sub-indices with PCA is included in the supplemental material.

**Table 1:** Selected causes for HAQ PCA.

**Non-Surgical HAQ Causes**
Epilepsy
Diabetes Mellitus
Chronic Kidney Disease
Adverse Effects of Medical Treatment
Cerebrovascular Disease
Hypertensive Heard Disease
Chronic Respiratory Diseases
Hodgkin Lymphoma
Leukemia
Neonatal Disorders
Tuberculosis
Diarrheal Diseases
Lower Respiratory Infections
Upper Respiratory Infections
Diphtheria
Whooping Cough
Tetanus
Measles
**Surgical HAQ Causes**
Congenital Heart Anomalies
Maternal Disorders
Non-Melanoma Skin Cancer (Squamous-Cell Carcinoma)
Cervical Cancer
Breast Cancer
Colon and Rectum Cancer
Uterine Cancer
Testicular Cancer
Rheumatic Heart Disease
Ischemic Heart Disease
Peptic Ulcer Disease
Appendicitis
Inguinal, Femoral, and Abdominal Hernia
Gallbladder and Biliary Diseases

The listed diseases are the causes included in the construction of the HAQ index. To construct the sub-indices, diseases were categorized by their primary treatment methodology. For the Non-surgical HAQ index, 18 causes were identified. For the Surgical HAQ index, 14 causes were identified.

### Analysis

Data from the World Bank, specifically Specialist Surgical Workforce Density and Number of Surgical Procedures per 100,000, were used to assist in validation of the PCA results. The methodology for this validation is included in the supplemental material.

In order to understand the improvement in surgical and non-surgical care in any specific country-year pair, a new variable, called relative improvement, was calculated. The relative improvement for each time period in each sub-index is calculated by subtracting the score in 1990 from the score in that time period. For example,

$$RI(y,c)=S_{sub-index}(y,c)-S_{sub-index}(1990,c),$$

where *RI*(*y*, *c*) represents the relative improvement for year *y* and country *c*, *S*_*sub−index*_(*y*, *c*) represents the sub-index score for year *y* and country *c*, and *S*_*sub−index*_(1990, *c*) represents the sub-index score for country c in 1990. Thus, for all countries, the relative improvement in 1990 is zero, and positive scores in subsequent time periods represent improvements over time.

Differences in scores between the relative surgical improvement and the relative non-surgical improvement represent different rates of change. We define improvement gap as the relative surgical improvement in 2016 minus the relative non-surgical improvement in 2016. For example,

$$G(c)=RI_{surgical}(2016,c)-RI_{non-surgical}(2016,c),$$

where *G*(*c*) represents the improvement gap in country *c*, *RI*_*surgical*_(2016, *c*) represents the relative surgical improvement in country *c* in 2016, and *RI*_*surgical*_(2016, *c*) represents the relative non-surgical improvement in country *c* in 2016. A positive improvement gap implies “better” improvement in access to quality surgical care for that country compared to the improvement in access to quality non-surgical care. S1 Fig presents a visual representation of these calculations in Cambodia.

A model was assessed in order to understand which factors contribute to the gap between surgical and non-surgical rate of change. The data in the model included government health expenditure per year as well as development assistance for health. Additionally, data was included on urbanization and GDP. All independent variables were standardized by subtracting the mean and dividing by the standard deviation. Using the plm package in R (R Core Team, 2019), a model was constructed using country fixed effects. This method removes country-level unobserved heterogeneity for any variables not included in the model. Data on expenditure was not available for 1990. Data on expenditure from 2015 was recoded to 2016 in order to allow for inclusion in the panel. Sensitivity analysis using the shorter panel showed no differences in the significance or signs of variables and coefficients. This regression included 137 of 195 countries, as complete data was not available for 58 countries. Model details are included in S1 and S2 Tables. All data was analyzed in Rstudio v3.5.1.

## Results

Both the surgical HAQ and non-surgical HAQ, which are scaled from 0–100, show similarly consistent upward trends over time. Globally, the surgical HAQ has increased 0.586 units per year from 1990–2016. The non-surgical HAQ, in aggregate, shows a 0.528 unit increase per year from 1990–2016 (S2 Fig). There has been a significant, yet small difference in the improvement of surgical HAQ score and non-surgical HAQ score over time (p < 0.05). There are consistently positive trends in both surgical HAQ and non-surgical HAQ in each income group and region (Table 2).

**Table 2:** Change of surgical HAQ and non-surgical HAQ over time by WB income group and GBD super region.

Group	Number of Countries	Coefficient (Surgical HAQ)	Coefficient (Non-Surgical HAQ)	Coefficient (Difference in Slopes)	95% Confidence Interval	Adjusted p-value (Difference in Slopes)
**World Bank Income Group**						
High Income	59	0.761	0.572	0.189	0.143, 0.236	<0.05
Upper Middle Income	53	0.717	0.559	0.160	0.101, 0.219	<0.05
Lower Middle Income	49	0.464	0.472	-0.007	-0.062, 0.048	1.00
Low Income	34	0.271	0.486	-0.214	-0.276, -0.152	<0.05
**GBD Super Region**						
Southeast Asia, East Asia, and Oceania	28	0.545	0.546	-0.001	-0.094, 0.093	1.00
Central Europe, Eastern Europe, and Central Asia	29	0.712	0.475	0.246	0.173, 0.32	<0.05
High-income	34	0.704	0.534	0.170	0.12, 0.22	<0.05
Latin America and Caribbean	32	0.677	0.502	0.175	0.116, 0.234	<0.05
North Africa and Middle East	21	0.752	0.736	0.014	-0.076, 0.104	1.00
South Asia	5	0.602	0.778	-0.176	-0.347, -0.005	0.532
Sub-Saharan Africa	46	0.309	0.431	-0.121	-0.178, -0.064	<0.05

For each WB income group and GBD super region, the change over time in both surgical HAQ and non-surgical HAQ was calculated. Also, difference in slopes models using country fixed effects were ran and coefficients of the difference in slopes presented. Positive coefficients correspond to groups where surgical HAQ improved more rapidly than non-surgical HAQ. Negative coefficients correspond to groups where non-surgical HAQ improved more rapidly than surgical HAQ. Adjusted p-values (using the bonferroni correction) are presented with a significance cutoff of 0.05.

**Fig 1:**
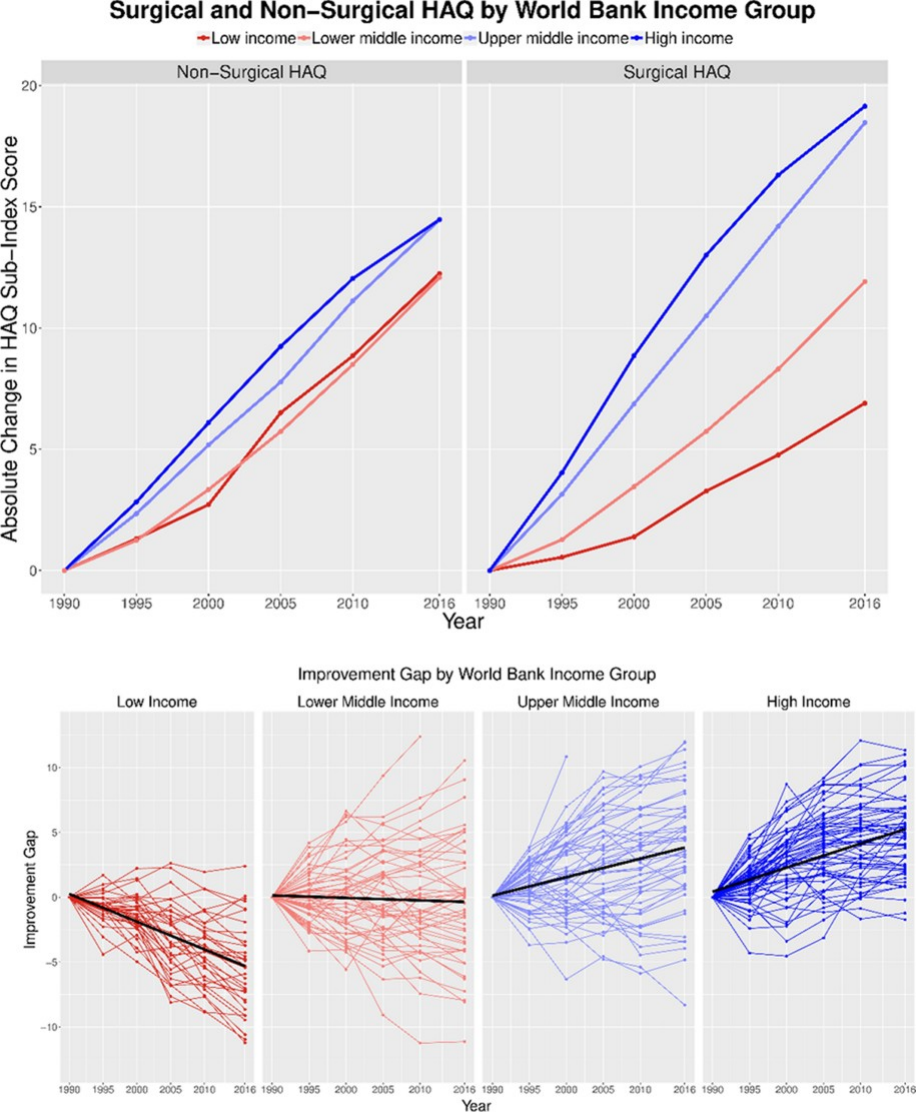
a: Surgical and Non-Surgical HAQ by World Bank Income Group. This figure depicts the relative progress in each sub-index by income group. While there has been little divergence between high- and low-income for the non-surgical sub-index, the surgical sub-index shows increased divergence over time. b: Improvement gap in surgical and non-surgical HAQ for each country, by WB income group. For each country-year pair, the relative change in non-surgical HAQ was subtracted from the relative change in surgical HAQ and plotted. A trend line in black is plotted through each group. Both low-income and high-income groups show increasing gap, though in different directions, with low-income countries on average having larger change over time for non-surgical HAQ than surgical HAQ.

**Fig 2:**
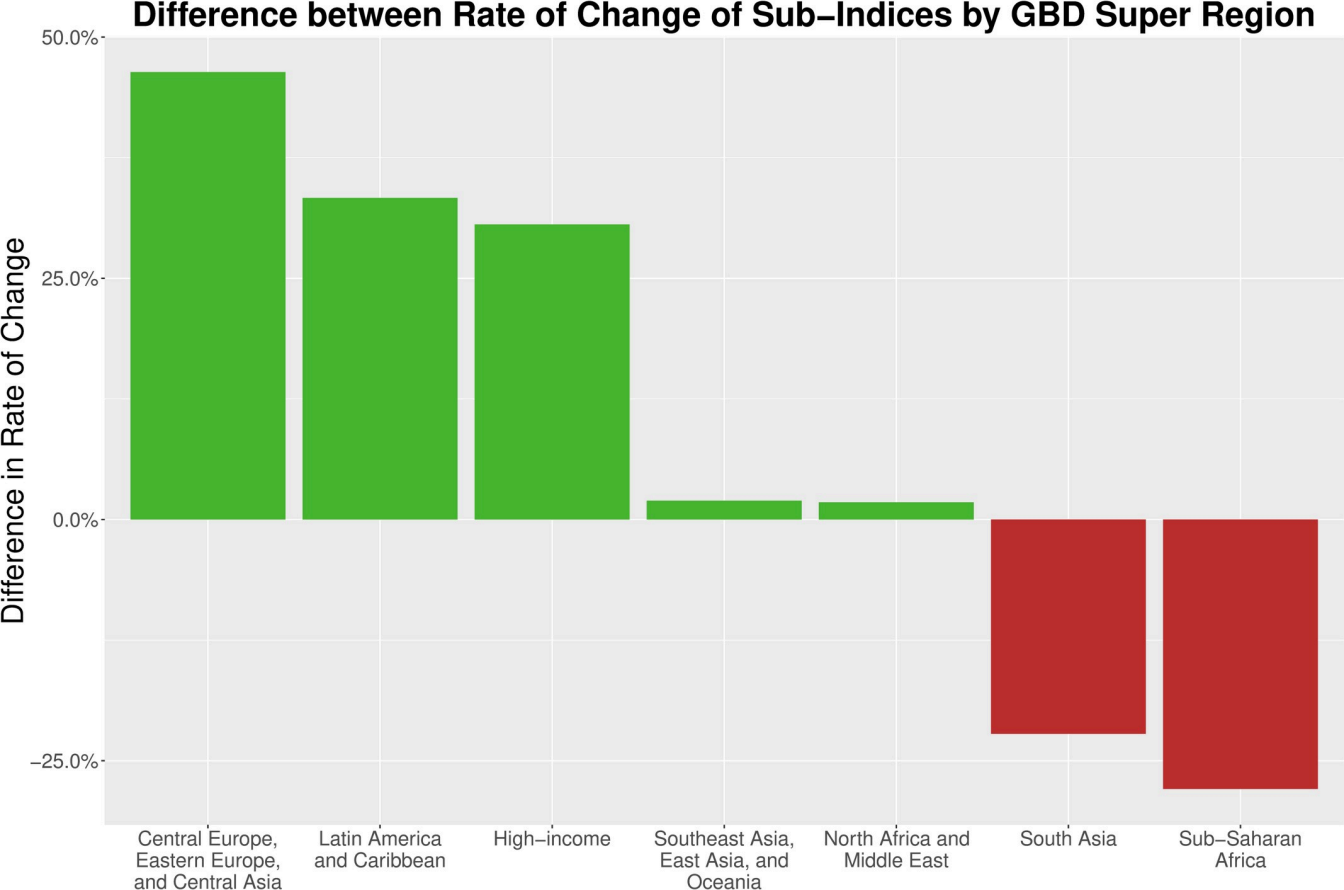
Difference in the rate of change of surgical and non-surgical sub-indices by GBD super region. This figure depicts the difference in the rate of change of the sub-indices by GBD Super Region. Green bars indicate that surgical HAQ has had more a more positive rate of change than non-surgical HAQ, and red bars indicate the opposite. See Table 2 for p-values of these changes.

While there is a small difference between surgical HAQ and non-surgical HAQ in aggregate, sub-analysis of income groups (Fig 1) and GBD super regions (Fig 2) show larger differences (Table 2). Low-income countries have experienced a significant difference between the slopes of surgical HAQ and non-surgical HAQ over time (p < 0.05), with surgical HAQ showing less improvement over time compared to non-surgical HAQ. High and upper-middle income countries have experienced a significant difference between the slopes of surgical HAQ and non-surgical HAQ over time (p < 0.05), with surgical HAQ showing better improvement over time compared to non-surgical HAQ (Table 2). Compared to low-income countries, high-income countries have been 2.77 times more effective at improving surgical HAQ, while they have been 1.18 times more effective at improving non-surgical HAQ.

There are regional differences in the relative rate of improvement between surgical and non-surgical HAQ. The Central Europe/Eastern Europe/Central Asia super region shows the largest difference in relative rate of improvement, with surgical HAQ improving 46.4% more rapidly than non-surgical HAQ (Fig 2). Sub-Saharan Africa shows a significantly different rate of improvement, with a 27.9% lower rate of improvement for surgical HAQ compared to non-surgical HAQ. The North Africa/Middle East, South Asia and Southeast Asia/East Asia/Oceania super regions show no significant differences in rate of improvement of the two sub-indices.

Three different country fixed effects models were run, using the same variables as independent variables (urbanization, GDP, development assistance for health, government expenditure on health) and three separate dependent variables (surgical HAQ, non-surgical HAQ, and both surgical and non-surgical HAQ as dummy variables). Government expenditure on health showed a significant positive relationship with improving both surgical HAQ (Surgical HAQ model, p < 0.05) and non-surgical HAQ (Non-Surgical HAQ model, p < 0.05), with a stronger effect in improving surgical HAQ (Table 3). In these two models, a one standard deviation increase in government expenditure for health is associated with a 3.999-point increase in surgical HAQ score, compared to a 2.241-point increase in non-surgical HAQ score. This association describes the average change over time within each country. Our models also show that development assistance for health has a significant positive relationship with improving non-surgical HAQ (Non-Surgical HAQ model, p < 0.05), while there is no significant effect on surgical HAQ (Surgical HAQ model, p = 0.24). A one standard deviation increase in development assistance for health is associated with a 0.998-point increase in non-surgical HAQ. The difference in slopes model confirms these findings, showing that development assistance for health is associated with more improvement in non-surgical HAQ versus surgical HAQ (p<0.05). It also shows that government expenditure for health is associated with more improvement in surgical HAQ versus non-surgical HAQ (p<0.05). GDP and urbanization were included in the model in order to control for economic changes in each country over time. This permits a more precise evaluation of development assistance for health and government expenditure on health. Magnitude of these coefficients should not be used comparatively, as higher coefficients do not necessarily mean greater importance beyond statistical significance.

**Table 3:** Results from panel regressions of HAQ sub-indices versus country funding indicators.

Variable	Surgical HAQ Model			Non-Surgical HAQ Model			Difference in Slopes Model		
	Coefficient	95% Confidence Interval	p-value	Coefficient	95% Confidence Interval	p-value	Coefficient	95% Confidence Interval	p-value
Development Assistance for Health, per capita	0.272	-0.185, 0.728	0.24	0.998	0.567, 1.428	<0.05	-1.142	-1.515, -0.769	<0.05
Government Expenditure on Health, per Capita	3.999	2.851, 5.147	<0.05	2.241	1.159, 3.322	<0.05	1.203	0.819, 1.587	<0.05
Urbanization	17.375	15.369, 19.382	<0.05	17.076	15.187, 18.966	<0.05	17.226	15.768, 18.684	<0.05
GDP per Capita	4.423	3.503, 5.344	<0.05	3.897	3.030, 4.763	<0.05	4.160	3.491, 4.829	<0.05

Three country fixed effect regressions were run with Surgical HAQ, Nonsurgical HAQ, and both Surgical HAQ and Non-Surgical HAQ as dummy variables. The surgical model shows that development assistance for health has no effect on Surgical HAQ, while the non-surgical model shows that it has a significant positive effect on Non-Surgical HAQ. Government expenditure on health significantly improves both Surgical HAQ and Non-Surgical HAQ. The difference in slopes model shows that government expenditure on health is associated with more improvement of Surgical HAQ versus Non-Surgical HAQ, whereas development assistance for health is associated with more improvement in Non-Surgical HAQ.

## Discussion

The IHME previously demonstrated that Healthcare Access and Quality (HAQ) is improving across most populations and socioeconomic groups. When subdivided into surgical and non-surgical HAQ sub-indices, overall improvements are seen in both at the global level. However, when comparing the surgical and non-surgical indices, it is clear that access to quality surgical care is lagging in low-income countries. Since 1990, the gap has widened for these poorer countries, partially driven by the more pronounced improvement in non-surgical care.

Our data suggest that an increased level of government expenditure on health has a stronger impact on the improvement of surgical HAQ versus non-surgical HAQ. In contrast, development assistance for health is only associated with the improvement of non-surgical HAQ. It is essential to view this finding through the historical lens of the global health investment strategy. External aid is rarely dedicated to health systems strengthening and is instead historically linked to vertical, disease-specific programming [17]. For example, funds for health sector support have not exceeded 23% of total aid since 1990, and the most recent data (2018) demonstrates a decrease to 14% [18]. Given the explicit focus on communicable disease and maternal/child health over the past 30 years, external aid acts to augment the gap in improvement by advancing care for non-surgical conditions without similar advances in surgical care. Although healthcare budgets are inadequate in most LMIC’s, a significant portion (about 1/3) is directed to building infrastructure and workforce for non-communicable diseases; the elements of a strong surgical system [19].

External funding, or development assistance for health, has prioritized communicable diseases and maternal/child health; programs that lend themselves to a more vertical approach and are relatively less reliant on health systems. Many of these programs have improved access and quality tremendously in a shorter time frame. Our findings suggest that the rapid improvement in non-surgical HAQ is, at least in part, influenced by such funding. Development assistance for health is often linked to global priorities, with funds tied to specific causes deemed important by the WHO or the UN. Setting an investment strategy has an appreciable effect. Care must be taken to ensure that priorities are aligned with disease burden.

Using surgical HAQ profiles provide an objective estimation of access and quality that can be used to track progress over time. HAQ measures allow for comparisons between countries, socioeconomic groups, and specific disease profiles. With the increasing interest from nations creating formal surgical plans, HAQ sub-indices could play a role in monitoring progress and evaluating interventions at the national level. These sub-indices would provide high-level data to complement current monitoring and evaluation frameworks and indicators. HAQ profiles also offer insights into the relative performance of different countries over time. LMICs with similar GDP, healthcare expenditure, and demographic profiles have vastly different HAQ sub-indices. For example, Nepal and Mali have very similar levels of development assistance for health from 1995 to 2010, even though Nepal had nearly double the difference in improvement between sub-indices. S4 Fig and S3 Table maps the relative performance of the sub-indices over time.

There are limitations of this study, both in its methodology and applicability. The data used for this study comes from modeled data on country and cause-specific mortality as opposed to primary data, which is currently not available. The Institute for Health Metrics and Evaluation provided the modeled epidemiological data used in this study, and uses transparent methodology and more than 90,000 data sources to generate estimates with appropriate levels of uncertainty [20]. Due to the lack of accurate surgical data for many countries, specifically LMICs, it is difficult to validate this new sub-index. A simple regression of specialist surgical workforce density and surgical volume against the surgical HAQ sub-index, included in S3 Fig, shows that the sub-index correlates with existing indicators. As more data is collected at the global level, this sub-index should be validated against other surgical indicators. The expenditure model should be interpreted carefully due to the methodology used. The model in this paper uses fixed country effects, which gives the average *within*-country change in the gap. Thus, the coefficients describe how the sub-indices change, on average, in each country. As the model does not encompass the entire time period studied due to lack of health expenditure data in 1990 and the need to recode 2015 data to 2016, this is a relatively short panel and should be interpreted appropriately. Finally, the use of the HAQ, surgical HAQ, and non-surgical HAQ indices to directly measure health system quality is inadvisable, as these indices function by measuring the sequelae of poor access to a quality medical system, rather than providing a direct measure of ‘quality’.

Healthcare access and quality sub-indices help measure dimensions of healthcare beyond quantity. This novel approach to measuring changing treatment modalities in healthcare systems permits studying how these systems change over time and how investment strategies impact outcomes for surgical and non-surgical disease. We found that significant gaps in improvement exist between surgical and non-surgical access and quality when using these sub-indices. Over the past 26 years, the gap in improvement between surgical and non-surgical HAQ has worsened in low-income countries, partially driven by differences in government expenditure for health and development assistance for health. Tracking temporal trends in HAQ provides an assessment of national health strategies, helping prioritization of future investments in healthcare. To that same end, case studies of specific nations with large or small gap in improvement over time would assist in discovering effective, existing models for health system strengthening. As more nations begin to understand the importance of surgery as part of their health infrastructure and begin to draft national surgical, obstetric, and anesthesia plans, this new metric will help guide and track progress towards achieving universal healthcare.

## Supporting information

S1 Fig Relative improvement example in Cambodia. In Cambodia, there has been a difference in the relative improvement of surgical HAQ and non-surgical HAQ. While surgical HAQ has improved in every time period, non-surgical HAQ has more than doubled the rate of improvement.
(TIF)

S2 Fig Relative change in surgical and non-surgical HAQ, aggregated. Each line represents the aggregate (all country) HAQ score for each specific sub-index. The rate of improvement is significantly different between the aggregate Non-Surgical and Surgical HAQ scores, though the difference is small. Calculation of p-value is based on a country fixed effect model with an interaction term between year and a dummy variable for sub-index.
(TIF)

S3 Fig Association of surgical HAQ with surgical workforce and number of surgical procedures. In order to validate the use of the Surgical HAQ index as an indicator for surgical quality, LCoGS indicators were used to form regressions for surgical indicators versus surgical HAQ. Logged surgical workforce data from 2014 are regressed against surgical HAQ from 2016. The model for surgical workforce has a positive slope (β = 10.729). Next, the most recent data for surgical volume (between 2010 and 2016), logged, was regressed against surgical HAQ in 2016. The model for surgical HAQ has a positive slope (β = 11.925). Overall, both surgical indicators show positive associations with surgical HAQ.
(TIF)

S4 Fig Map of improvement gap by country. Countries on the green end of the spectrum have positive improvement gaps between 1990 and 2016. Countries on the red end of the spectrum have negative improvement gaps. A positive improvement gap indicates that surgical HAQ has had a more positive rate of change than non-surgical HAQ. The majority of countries with negative improvement gaps (where surgical HAQ was outpaced by non-surgical HAQ) are located in Africa and Asia.
(TIF)

S1 Table Surgical and non-surgical models. Two models were constructed using the same independent variables with different dependent variables. Development assistance for health and governmental health expenditure per capita were the predictors, controlled by urbanization rate and GDP per capita. Country fixed effects were used to remove country-level unobserved heterogeneity for any variables not included in the model. Model 1 includes only Surgical HAQ as the dependent variable. Model 2 includes only Non-Surgical HAQ as the dependent variable.
(DOCX)

S2 Table Interaction models. An additional model was constructed, Model 3, which uses interactions of HAQ sub-index type with the variables of interest to show the difference in slopes. A positive coefficient in the interaction term signifies a difference in improvement that favors Non-surgical HAQ for that explanatory variable. A negative coefficient in the interaction term signifies a difference in improvement that favors Surgical HAQ for that explanatory variable. eTable 1 and 2 give the details of each regression. Bold variables indicate significance below p = 0.001.
(DOCX)

S3 Table Countries with the highest and lowest change in improvement gap over 26 years. Countries were ranked based on their improvement gap. The improvement gap is calculated by subtracting the relative improvement in non-surgical HAQ from the relative improvement in surgical HAQ, from 1990 to 2016.
(DOCX)

S1 Appendix Additional information on calculation of PCA indices. (DOCX)

## References

[1] Mortality. World Population Prospects, The 2019 Revision—Volume I: Comprehensive Tables. 6 2019:180–181. 10.18356/c8776a5c-en

[2] WHO, UNICEF, UNFPA, World Bank Group, and United Nations Population Division. Trends in Maternal Mortality: 1990 to 2015 Geneva: World Health Organization, 2015. Population and Development Review. 2015;42(4):15–15. 10.1016/S0140-6736(15)00838-7 26584737 PMC5515236

[3] You DR, Hug LR, Ejdemyr SR, et al Global, regional, and national levels and trends in under-5 mortality between 1990 and 2015, with scenario-based projections to 2030: a systematic analysis by the UN Inter-agency Group for Child Mortality Estimation. The Lancet. 2015;386(10010):2275–2286. 10.1016/s0140-6736(15)00120-8 26361942

[4] Williams J, Allen L, Wickramasinghe K, Mikkelsen B, Roberts N, Townsend N. A systematic review of associations between non-communicable diseases and socioeconomic status within low- and lower-middle-income countries. Journal of Global Health. 2018;8(2). 10.7189/jogh.08.020409 30140435 PMC6076564

[5] Scott A, Lucci P, Berliner T. Mind the Gap? A Comparison of International and National Targets for the SDG Agenda. London: Overseas Development Institute; 2015.

[6] Souza JAD, Hunt B, Asirwa FC, Adebamowo C, Lopes G. Global Health Equity: Cancer Care Outcome Disparities in High-, Middle-, and Low-Income Countries. Journal of Clinical Oncology. 2016;34(1):6–13. 10.1200/JCO.2015.62.2860 26578608 PMC5795715

[7] Engelgau MM, Sampson UK, Rabadan-Diehl C, et al Tackling NCD in LMIC. Glob Heart. 2016; 11(1): 5–15. 10.1016/j.gheart.2015.12.016 27102018 PMC4843818

[8] Dieleman JL, Graves C, Johnson E, et al Sources and Focus of Health Development Assistance, 1990–2014. Jama. 2015;313(23):2359–2368. 10.1001/jama.2015.5825 26080340

[9] Meara JG, Leather AJ, Hagander L, et al Global Surgery 2030: evidence and solutions for achieving health, welfare, and economic development. International Journal of Obstetric Anesthesia. 2016;25:75–78. 10.1016/j.ijoa.2015.09.006 26597405

[10] Shrime MG, Bickler SW, Alkire BC, Mock C. Global burden of surgical disease: an estimation from the provider perspective. The Lancet Global Health. 2015;3 10.1016/S2214-109X(14)70384-5 25926322

[11] Alkire BC, Bekele A, Citron I, et al Building capacity for surgery, obstetrics and anesthesia in support of universal health coverage and achievement of the Sustainable Development Goals. East and Central African Journal of Surgery. 2019; 24(1): 3–8.

[12] Alkire BC, Peters AW, Shrime MG, Meara JG. The Economic Consequences Of Mortality Amenable To High-Quality Health Care In Low- And Middle-Income Countries. Health Affairs. 2018;37(6):988–996. 10.1377/hlthaff.2017.1233 29863936

[13] Kruk ME, Gage AD, Joseph NT, Danaei G, García-Saisó S, Salomon JA. Mortality due to low-quality health systems in the universal health coverage era: a systematic analysis of amenable deaths in 137 countries. The Lancet. 2018;392(10160):2203–2212. 10.1016/S0140-6736(18)31668-4 30195398 PMC6238021

[14] National Academies of Sciences, Engineering, and Medicine, Health and Medicine Division, Board on Health Care Services, Board on Global Health, Committee on Improving the Quality of Health Care Globally. Crossing the Global Quality Chasm: Improving Health Care Worldwide. Washington (DC), 2018.

[15] GBD 2015 Healthcare Access and Quality Collaborators. Healthcare Access and Quality Index based on mortality from causes amenable to personal health care in 195 countries and territories, 1990–2015: a novel analysis from the Global Burden of Disease Study 2015. Lancet. 2017; 390(10091): 231–66. 10.1016/S0140-6736(17)30818-8 28528753 PMC5528124

[16] Global Burden of Disease Collaborative Network. Global Health Spending 1995–2015. Seattle, United States: Institute for Health Metrics and Evaluation (IHME), 2018.

[17] Dieleman JL, Schneider MT, Haakenstad A, Singh L, Sadat N, Birger M, et al Development assistance for health: past trends, associations, and the future of international financial flows for health. The Lancet. 2016 6 18;387(10037):2536–44. 10.1016/S0140-6736(16)30168-4 27086170

[18] Institute for Health Metrics and Evaluation (IHME). Development Assistance for Health Database 1990–2018. Seattle, United States: Institute for Health Metrics and Evaluation (IHME), 2019.

[19] Xu K, Soucat A & Kutzin J, et al Public Spending on Health: A Closer Look at Global Trends. Geneva: World Health Organization; 2018 (WHO/HIS/HGF/HFWorkingPaper/18.3).

[20] Gall J, McKee S, Redford S. healthdata.org. healthdataorg. October 2018. http://www.healthdata.org/acting-data/power-models. Accessed January 1, 2020.

