# Supplementary figures and images for: A country-level comparison of access to quality surgical and non-surgical healthcare from 1990-2016
Source: PLoS One. 2020 Nov 3;15(11):e0241669. doi: 10.1371/journal.pone.0241669 (PMC7608906; doi:10.1371/journal.pone.0241669)

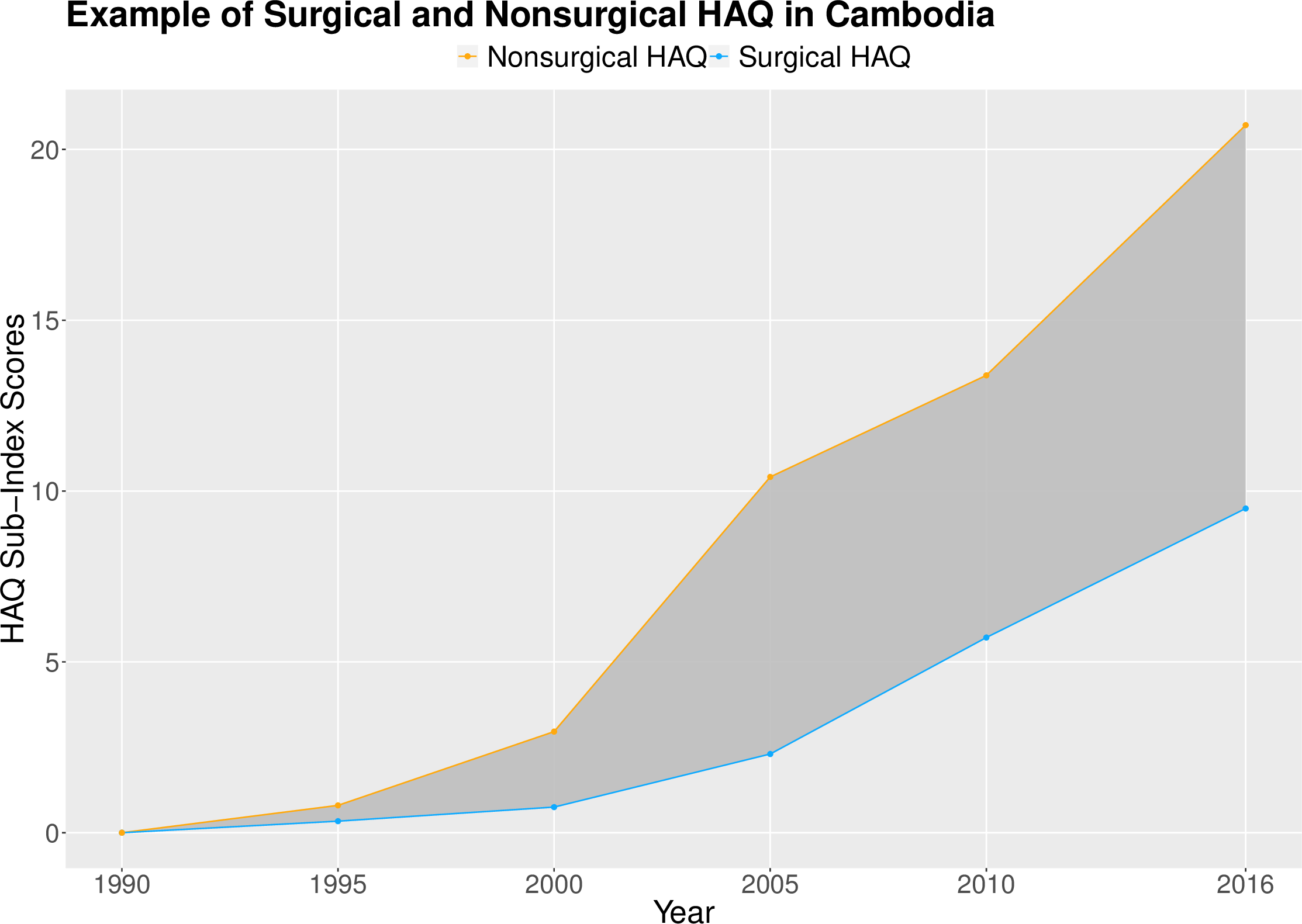

Supplement: S1 Fig — In Cambodia, there has been a difference in the relative improvement of surgical HAQ and non-surgical HAQ. While surgical HAQ has improved in every time period, non-surgical HAQ has more than doubled the rate of improvement. (TIF) [file pone.0241669.s001.tif]

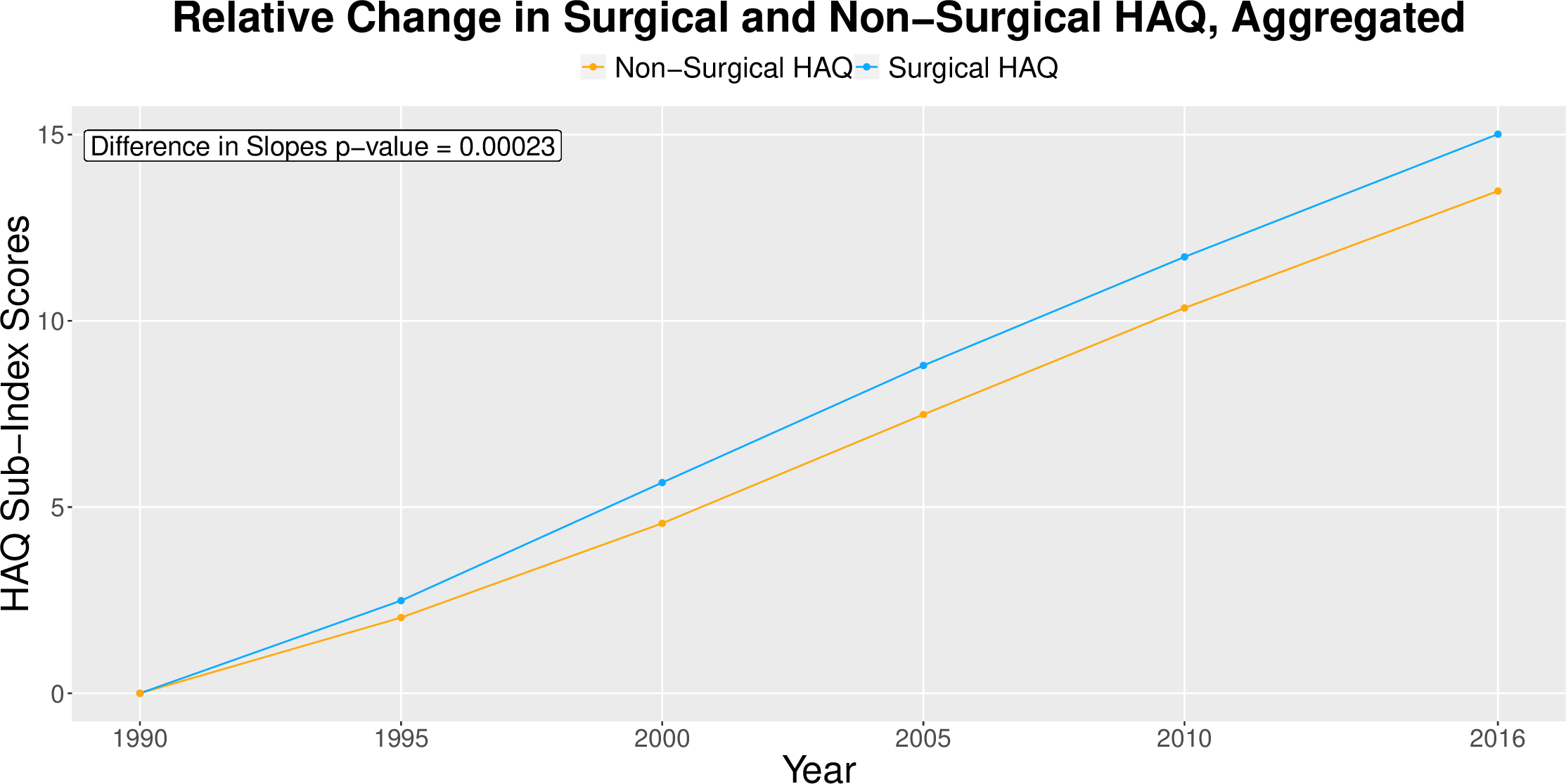

Supplement: S2 Fig — Each line represents the aggregate (all country) HAQ score for each specific sub-index. The rate of improvement is significantly different between the aggregate Non-Surgical and Surgical HAQ scores, though the difference is small. Calculation of p-value is based on a country fixed effect model with an interaction term between year and a dummy variable for sub-index. (TIF) [file pone.0241669.s002.tif]

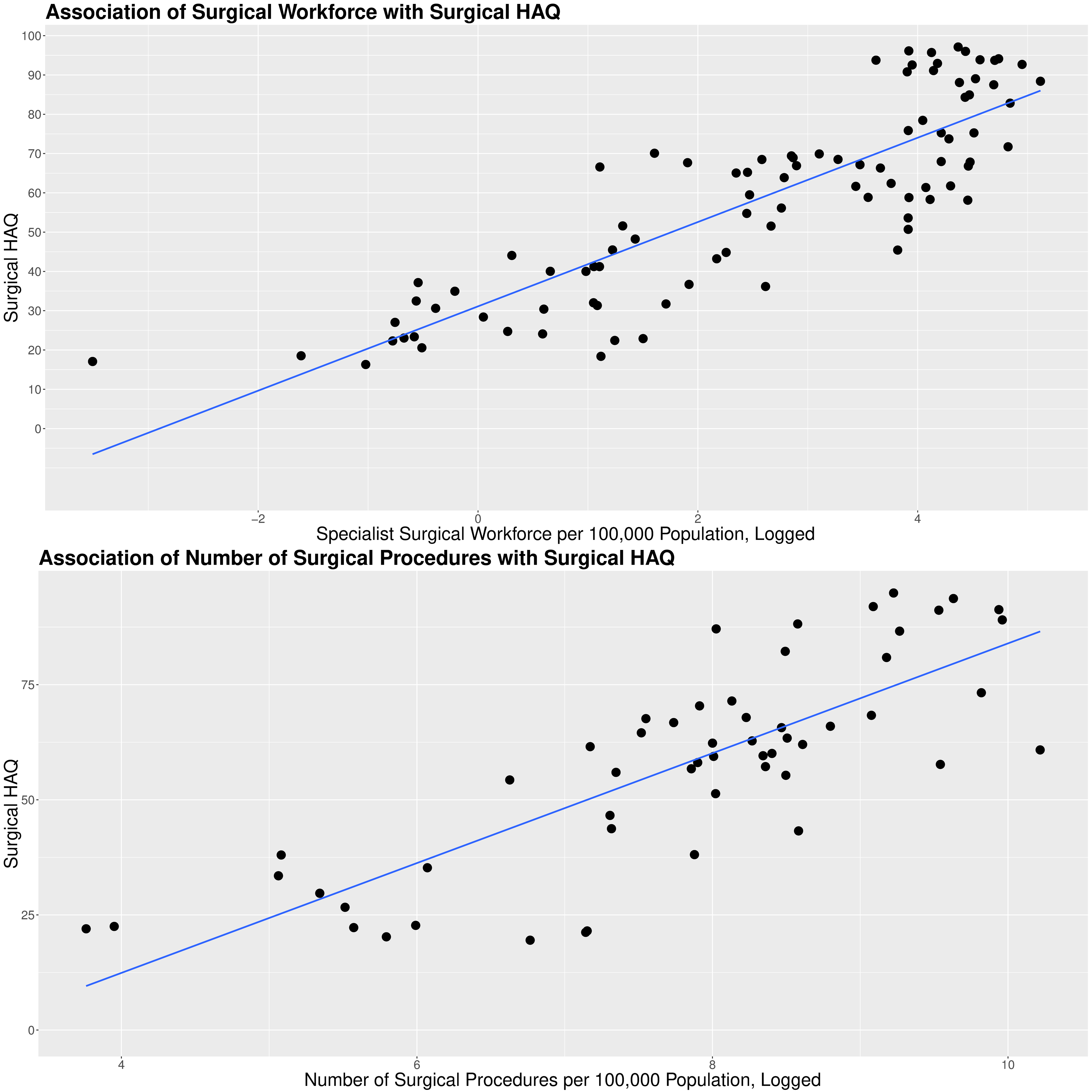

Supplement: S3 Fig — In order to validate the use of the Surgical HAQ index as an indicator for surgical quality, LCoGS indicators were used to form regressions for surgical indicators versus surgical HAQ. Logged surgical workforce data from 2014 are regressed against surgical HAQ from 2016. The model for surgical workforce has a positive slope (β = 10.729). Next, the most recent data for surgical volume (between 2010 and 2016), logged, was regressed against surgical HAQ in 2016. The model for surgical HAQ has a positive slope (β = 11.925). Overall, both surgical indicators show positive associations with surgical HAQ. (TIF) [file pone.0241669.s003.tif]

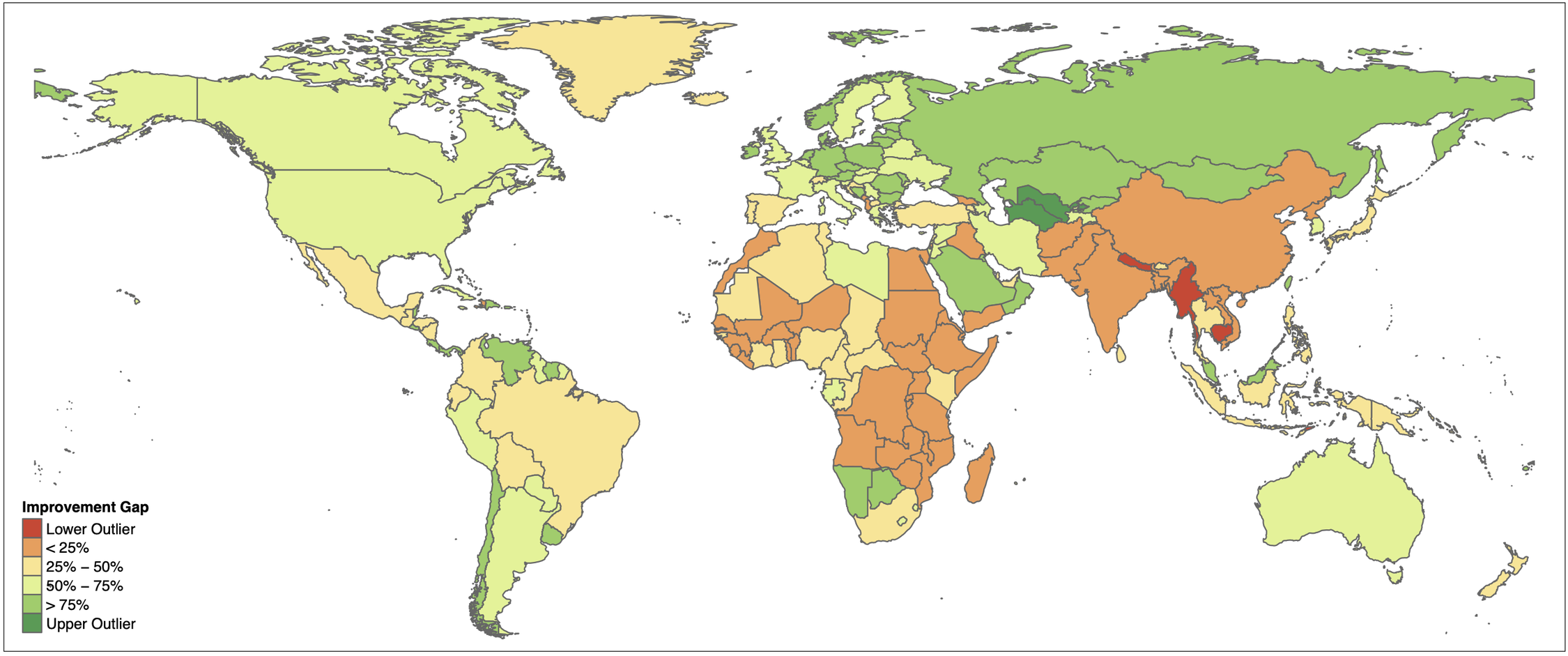

Supplement: S4 Fig — Countries on the green end of the spectrum have positive improvement gaps between 1990 and 2016. Countries on the red end of the spectrum have negative improvement gaps. A positive improvement gap indicates that surgical HAQ has had a more positive rate of change than non-surgical HAQ. The majority of countries with negative improvement gaps (where surgical HAQ was outpaced by non-surgical HAQ) are located in Africa and Asia. (TIF) [file pone.0241669.s004.tif]
