# Supplementary material for: A country-level comparison of access to quality surgical and non-surgical healthcare from 1990-2016
Source: PLoS One. 2020 Nov 3;15(11):e0241669. doi: 10.1371/journal.pone.0241669 (PMC7608906; doi:10.1371/journal.pone.0241669)
Supplement: S1 Table — Two models were constructed using the same independent variables with different dependent variables. Development assistance for health and governmental health expenditure per capita were the predictors, controlled by urbanization rate and GDP per capita. Country fixed effects were used to remove country-level unobserved heterogeneity for any variables not included in the model. Model 1 includes only Surgical HAQ as the dependent variable. Model 2 includes only Non-Surgical HAQ as the dependent variable. (DOCX) [file pone.0241669.s005.docx]

**Supplemental Table 1: Surgical and Non-Surgical Models**

|  | **Model 1** | **Model 2** |
| --- | --- | --- |
| Dependent Variable | Surgical HAQ | Non-Surgical HAQ |
| Independent Variables |  |  |
| *Development Assistance for Health, Coef (t-value)* | 0.27150 (1.1646) | **0.99763 (4.5439)** |
| *Governmental Health Expenditure per Capita, Coef (t-value)* | **3.99889 (6.8255)** | **2.24063 (4.0611)** |
| *Urbanization Rate, Coef (t-value)* | **17.37519 (16.9729)** | **17.07636 (17.7132)** |
| *GDP per Capita, Coef (t-value)* | **4.42318 (9.4180)** | **3.89657 (8.8101)** |
| Total number of observations (Number of countries times number of time periods) | 683 | 683 |
| R-Squared (adjusted) | 0.64116 (0.54848) | 0.62626 (0.52973) |
| F-statistic | 242.11 | 227.055 |

*S1 Table 1: Two models were constructed using the same independent variables with different dependent variables. Development assistance for health and governmental health expenditure per capita were the predictors, controlled by urbanization rate and GDP per capita. Country fixed effects were used to remove country-level unobserved heterogeneity for any variables not included in the model. Model 1 includes only Surgical HAQ as the dependent variable. Model 2 includes only Non-Surgical HAQ as the dependent variable.*
