# Supplementary material for: A country-level comparison of access to quality surgical and non-surgical healthcare from 1990-2016
Source: PLoS One. 2020 Nov 3;15(11):e0241669. doi: 10.1371/journal.pone.0241669 (PMC7608906; doi:10.1371/journal.pone.0241669)
Supplement: S2 Table — An additional model was constructed, Model 3, which uses interactions of HAQ sub-index type with the variables of interest to show the difference in slopes. A positive coefficient in the interaction term signifies a difference in improvement that favors Non-surgical HAQ for that explanatory variable. A negative coefficient in the interaction term signifies a difference in improvement that favors Surgical HAQ for that explanatory variable. eTable 1 and 2 give the details of each regression. Bold variables indicate significance below p = 0.001. (DOCX) [file pone.0241669.s006.docx]

**Supplemental Table 2: Interaction Model**

|  | **Model 3** |
| --- | --- |
| Dependent Variable | HAQ Sub-index Score |
| Independent Variables |  |
| *Development Assistance for Health, Coef (t-value)* | **1.20550 (6.2048)** |
| *Governmental Health Expenditure per Capita, Coef (t-value)* | **2.51827 (5.7651)** |
| *Urbanization Rate, Coef (t-value)* | **17.22578 (23.1588)** |
| *GDP per Capita, Coef (t-value)* | **4.15987 (12.1903)** |
| *Development Assistance for Health interaction with HAQ subindex, Coef (t-value)* | **-1.14187 (-6.0009)** |
| *Governmental Health Expenditure per Capita interaction with HAQ subindex, Coef (t-value)* | **1.20298 (6.1418)** |
| *HAQ subindex* | **1.07929 (5.4574)** |
| Total number of observations (Number of countries times number of time periods) | 1366 |
| R-Squared (adjusted) | 0.59539 (0.54804) |
| F-statistic | 256.883 |

*S2 Table: An additional model was constructed, Model 3, which uses interactions of HAQ sub-index type with the variables of interest to show the difference in slopes. A positive coefficient in the interaction term signifies a difference in improvement that favors Non-surgical HAQ for that explanatory variable. A negative coefficient in the interaction term signifies a difference in improvement that favors Surgical HAQ for that explanatory variable. eTable 1 and 2 give the details of each regression. Bold variables indicate significance below p = 0.001.*
