# Supplementary material for: A country-level comparison of access to quality surgical and non-surgical healthcare from 1990-2016
Source: PLoS One. 2020 Nov 3;15(11):e0241669. doi: 10.1371/journal.pone.0241669 (PMC7608906; doi:10.1371/journal.pone.0241669)
Supplement: S3 Table — Countries were ranked based on their improvement gap. The improvement gap is calculated by subtracting the relative improvement in non-surgical HAQ from the relative improvement in surgical HAQ, from 1990 to 2016. (DOCX) [file pone.0241669.s007.docx]

**Supplemental Table 3: Countries with the Highest and Lowest Improvement Gap over 26 Years**

| **Country** | **Surgical HAQ** | **Non-Surgical HAQ** | **Improvement Gap** |
| --- | --- | --- | --- |
| Cambodia | 9.5 | 20.7 | -11.2 |
| Timor-Leste | 11.8 | 23 | -11.2 |
| Nepal | 10.1 | 21 | -10.9 |
| Myanmar (Burma) | 12.8 | 23.4 | -10.6 |
| Niger | 3.9 | 13.4 | -9.5 |
| Congo - Kinshasa | 0.9 | 10 | -9.1 |
| Liberia | 3.4 | 12.5 | -9.1 |
| Ethiopia | 11 | 19.7 | -8.7 |
| Angola | 7 | 15.3 | -8.3 |
| Laos | 10.4 | 18.5 | -8.1 |
| Afghanistan | 2.3 | 10.3 | -8 |
| Yemen | 10.6 | 18.6 | -8 |
| Eritrea | 8.5 | 16.4 | -7.9 |
| North Korea | -2.5 | 4.8 | -7.3 |
| Burkina Faso | 5.8 | 13 | -7.2 |
| South Sudan | -0.3 | 6.9 | -7.2 |
| Rwanda | 13.8 | 20.9 | -7.1 |
| Sierra Leone | 2.8 | 9.9 | -7.1 |
| Haiti | 9.4 | 16 | -6.6 |
| Sudan | 10.9 | 17.2 | -6.3 |
| Zimbabwe | -11.2 | -5 | -6.2 |
| Zambia | 1.3 | 7.4 | -6.1 |
| Burundi | 9.1 | 15 | -5.9 |
| Guinea | 3.1 | 8.5 | -5.4 |
| Mali | 11.8 | 17.1 | -5.3 |
| Senegal | 3 | 8.1 | -5.1 |
| Vietnam | 18.5 | 23.6 | -5.1 |
| Mozambique | 11.5 | 16.4 | -4.9 |
| Somalia | 2.7 | 7.6 | -4.9 |
| Maldives | 30.6 | 35.4 | -4.8 |
| Madagascar | 5.1 | 9.8 | -4.7 |
| Benin | 5.8 | 10.3 | -4.5 |
| Uganda | 8.5 | 12.8 | -4.3 |
| India | 11.6 | 15.7 | -4.1 |
| Pakistan | 6.1 | 10.2 | -4.1 |
| Sao Tome and Principe | 8.9 | 13 | -4.1 |
| **Country** | **Surgical HAQ** | **Non-Surgical HAQ** | **Disparity** |
| Egypt | 18.5 | 22.5 | -4 |
| Iraq | 5.5 | 9.5 | -4 |
| Tanzania | 8.4 | 12.1 | -3.7 |
| Togo | 5.5 | 9.2 | -3.7 |
| Georgia | 3.8 | 7.4 | -3.6 |
| China | 31.3 | 34.7 | -3.4 |
| Djibouti | 8.4 | 11.8 | -3.4 |
| Malawi | 9.8 | 13.2 | -3.4 |
| Montenegro | 9.7 | 13.1 | -3.4 |
| Morocco | 15.4 | 18.5 | -3.1 |
| Albania | 16.7 | 19.7 | -3 |
| Bangladesh | 24.9 | 27.6 | -2.7 |
| Palestinian Territories | 6.2 | 8.8 | -2.6 |
| Ghana | 8.7 | 11.1 | -2.4 |
| Congo - Brazzaville | 10.9 | 12.9 | -2 |
| Guinea-Bissau | 7.1 | 9 | -1.9 |
| Japan | 14.1 | 15.8 | -1.7 |
| Bolivia | 19.7 | 21.3 | -1.6 |
| Cote d’Ivoire | 4 | 5.6 | -1.6 |
| Cameroon | 4.4 | 5.9 | -1.5 |
| Turkey | 29.2 | 30.5 | -1.3 |
| Honduras | 15.6 | 16.8 | -1.2 |
| Macedonia | 15.2 | 16.4 | -1.2 |
| Qatar | 23.9 | 25.1 | -1.2 |
| Mexico | 17.7 | 18.7 | -1 |
| Central African Republic | 1.5 | 2.4 | -0.9 |
| Chad | 4.3 | 5.2 | -0.9 |
| Nicaragua | 15.7 | 16.6 | -0.9 |
| Papua New Guinea | 7.1 | 7.6 | -0.5 |
| Gambia | 6.7 | 7 | -0.3 |
| Equatorial Guinea | 33.1 | 33.3 | -0.2 |
| Bhutan | 24.2 | 24.3 | -0.1 |
| Kenya | 5.6 | 5.7 | -0.1 |
| Armenia | 15.2 | 15.2 | 0 |
| Comoros | 12.8 | 12.7 | 0.1 |
| Guatemala | 17.9 | 17.7 | 0.2 |
| Nigeria | 12.6 | 12.4 | 0.2 |
| Ecuador | 22.2 | 21.9 | 0.3 |
| **Country** | **Surgical HAQ** | **Non-Surgical HAQ** | **Disparity** |
| Indonesia | 14 | 13.7 | 0.3 |
| Kiribati | 5.4 | 5.1 | 0.3 |
| Philippines | 9.6 | 9.2 | 0.4 |
| Mauritania | 14.2 | 13.6 | 0.6 |
| Solomon Islands | 4.3 | 3.7 | 0.6 |
| South Africa | 8.4 | 7.8 | 0.6 |
| Sri Lanka | 20.5 | 19.9 | 0.6 |
| Thailand | 23.5 | 22.8 | 0.7 |
| Croatia | 14.7 | 13.9 | 0.8 |
| Greenland | 14.6 | 13.8 | 0.8 |
| Lebanon | 34.1 | 33.3 | 0.8 |
| New Zealand | 15 | 14.2 | 0.8 |
| Spain | 18.4 | 17.6 | 0.8 |
| Brazil | 17.1 | 16.2 | 0.9 |
| Colombia | 20.5 | 19.5 | 1 |
| Switzerland | 11 | 9.8 | 1.2 |
| Andorra | 11 | 9.5 | 1.5 |
| Iceland | 12.7 | 10.9 | 1.8 |
| Portugal | 21.2 | 19.3 | 1.9 |
| Tunisia | 21.9 | 20 | 1.9 |
| Algeria | 20 | 18 | 2 |
| Northern Mariana Islands | 12.9 | 10.9 | 2 |
| Israel | 17.1 | 15 | 2.1 |
| Serbia | 14.1 | 12 | 2.1 |
| United Arab Emirates | 19.9 | 17.8 | 2.1 |
| United States | 9.3 | 7.2 | 2.1 |
| Jamaica | 10.9 | 8.6 | 2.3 |
| South Korea | 33.6 | 31.3 | 2.3 |
| Syria | 22.1 | 19.8 | 2.3 |
| Italy | 16.7 | 14.3 | 2.4 |
| Peru | 26.1 | 23.7 | 2.4 |
| Tajikistan | 10.1 | 7.7 | 2.4 |
| Greece | 13.9 | 11.4 | 2.5 |
| Luxembourg | 18 | 15.5 | 2.5 |
| Samoa | 11 | 8.4 | 2.6 |
| Cape Verde | 15.7 | 13 | 2.7 |
| Tonga | 11 | 8.3 | 2.7 |
| Finland | 18.8 | 15.7 | 3.1 |
| **Country** | **Surgical HAQ** | **Non-Surgical HAQ** | **Disparity** |
| Gabon | 13.5 | 10.4 | 3.1 |
| U.S. Virgin Islands | 18.5 | 15.4 | 3.1 |
| Cuba | 14.3 | 11 | 3.3 |
| Jordan | 21.4 | 18.1 | 3.3 |
| Slovenia | 20.8 | 17.5 | 3.3 |
| Vanuatu | 4.6 | 1.1 | 3.5 |
| Argentina | 16.4 | 12.8 | 3.6 |
| Marshall Islands | 11.1 | 7.5 | 3.6 |
| Micronesia (Federated States of) | 13.7 | 10.1 | 3.6 |
| Belgium | 16.3 | 12.5 | 3.8 |
| Bahamas | 12.7 | 8.7 | 4 |
| Bermuda | 24.8 | 20.8 | 4 |
| Slovakia | 19 | 14.9 | 4.1 |
| Paraguay | 12.9 | 8.6 | 4.3 |
| Iran | 23 | 18.6 | 4.4 |
| Azerbaijan | 16.9 | 12.4 | 4.5 |
| Hungary | 19.5 | 15 | 4.5 |
| Sweden | 14.4 | 9.9 | 4.5 |
| Seychelles | 20.6 | 15.9 | 4.7 |
| United Kingdom | 17.2 | 12.5 | 4.7 |
| France | 17.9 | 13.1 | 4.8 |
| Guam | 8.8 | 4 | 4.8 |
| Cyprus | 26.3 | 21.4 | 4.9 |
| Barbados | 14.2 | 9.2 | 5 |
| Belarus | 17.4 | 12.4 | 5 |
| Lesotho | 2.7 | -2.3 | 5 |
| Ukraine | 12.5 | 7.5 | 5 |
| American Samoa | 14 | 8.9 | 5.1 |
| Australia | 17 | 11.7 | 5.3 |
| Bahrain | 24.9 | 19.6 | 5.3 |
| Canada | 15 | 9.7 | 5.3 |
| Guyana | 13.6 | 8.3 | 5.3 |
| Libya | 22.5 | 17.2 | 5.3 |
| Swaziland | 9.6 | 4.3 | 5.3 |
| Namibia | 19.3 | 13.9 | 5.4 |
| Austria | 17.8 | 12.3 | 5.5 |
| Singapore | 26.9 | 21.4 | 5.5 |
| Suriname | 14.1 | 8.6 | 5.5 |
| **Country** | **Surgical HAQ** | **Non-Surgical HAQ** | **Disparity** |
| Antigua & Barbuda | 16.3 | 10.7 | 5.6 |
| Kyrgyzstan | 12.4 | 6.8 | 5.6 |
| Saudi Arabia | 29.5 | 23.9 | 5.6 |
| Brunei | 17.2 | 11.5 | 5.7 |
| Oman | 25.9 | 19.8 | 6.1 |
| Botswana | 16.7 | 10.5 | 6.2 |
| Costa Rica | 16.9 | 10.7 | 6.2 |
| Uruguay | 17.4 | 11.2 | 6.2 |
| Ireland | 24.2 | 17.9 | 6.3 |
| Kuwait | 18.4 | 11.9 | 6.5 |
| Lithuania | 15.3 | 8.8 | 6.5 |
| Belize | 12 | 5.4 | 6.6 |
| Venezuela | 19.4 | 12.8 | 6.6 |
| Bulgaria | 15.9 | 9.2 | 6.7 |
| Norway | 18.3 | 11.5 | 6.8 |
| Trinidad & Tobago | 17.3 | 10.5 | 6.8 |
| Russia | 16 | 9.1 | 6.9 |
| Malaysia | 26.6 | 19.6 | 7 |
| Malta | 20.7 | 13.7 | 7 |
| Estonia | 22.6 | 15.2 | 7.4 |
| Puerto Rico | 20.7 | 13.2 | 7.5 |
| El Salvador | 27.5 | 19.8 | 7.7 |
| Netherlands | 18.1 | 10.4 | 7.7 |
| Dominican Republic | 25.3 | 17.3 | 8 |
| Kazakhstan | 17.4 | 9.4 | 8 |
| Fiji | 10.6 | 2.4 | 8.2 |
| Moldova | 15.4 | 6.4 | 9 |
| Romania | 24.9 | 15.9 | 9 |
| St. Vincent & Grenadines | 12.5 | 3.5 | 9 |
| Taiwan | 29.9 | 20.8 | 9.1 |
| Czechia | 23.9 | 14.6 | 9.3 |
| Bosnia & Herzegovina | 24.9 | 15.5 | 9.4 |
| Mauritius | 20.8 | 10.8 | 10 |
| Chile | 28.2 | 18 | 10.2 |
| Latvia | 19.2 | 9 | 10.2 |
| St. Lucia | 20.2 | 9.8 | 10.4 |
| Germany | 20.6 | 10.1 | 10.5 |
| Mongolia | 22.4 | 11.8 | 10.6 |
| **Country** | **Surgical HAQ** | **Non-Surgical HAQ** | **Disparity** |
| Denmark | 18.5 | 7.5 | 11 |
| Grenada | 16.5 | 5.1 | 11.4 |
| Poland | 29.2 | 17.8 | 11.4 |
| Dominica | 15.4 | 3.5 | 11.9 |
| Panama | 22.2 | 10.2 | 12 |
| Uzbekistan | 18.4 | 5.1 | 13.3 |
| Turkmenistan | 24.1 | 7.2 | 16.9 |

*S3 Table: Countries were ranked based on their improvement gap. The improvement gap is calculated by subtracting the relative improvement in non-surgical HAQ from the relative improvement in surgical HAQ, from 1990 to 2016.*
