# Supplementary material for: A country-level comparison of access to quality surgical and non-surgical healthcare from 1990-2016
Source: PLoS One. 2020 Nov 3;15(11):e0241669. doi: 10.1371/journal.pone.0241669 (PMC7608906; doi:10.1371/journal.pone.0241669)

**S1 Appendix**

**PCA Calculation**

The construction of the original IHME HAQ index is derived from 32 causes of mortality known to be amenable to high-quality healthcare. Estimates of mortality amenable to healthcare for each condition by country are ranked and re-scaled from 0-100, with relatively lower mortality rates resulting in a higher score. Using these cause, country, and time specific scores for the 32 conditions, principal components analysis (PCA) was utilized to build 2 sub-indices: surgical HAQ and non-surgical HAQ. The PCA technique is used to take a set of variables and reduce them to their ‘principal components’, which allows for calculation of sub-indexes through deriving variable weights. For each sub-index, the principal components that explained >80% of the variance were determined for each sub-index; two principal components for the surgical HAQ and four for non-surgical HAQ were used. PCA weights for each cause, which were derived from each specific principal component, are used in the calculation for the final surgical and non-surgical HAQ scores. Each cause-specific PCA weight from each principal component was itself weighted by the proportional variance explained by that principal component, and then added together. These scores are then rescaled to a scale of 0-1. These scaled weights for each cause are then multiplied by the cause specific HAQ score for each country. The formula for each calculation is included in the below. The transformed cause specific HAQ scores for each country are added to construct the final surgical and non-surgical HAQ scores. These sub-indices were calculated for each country-year available in HAQ index.


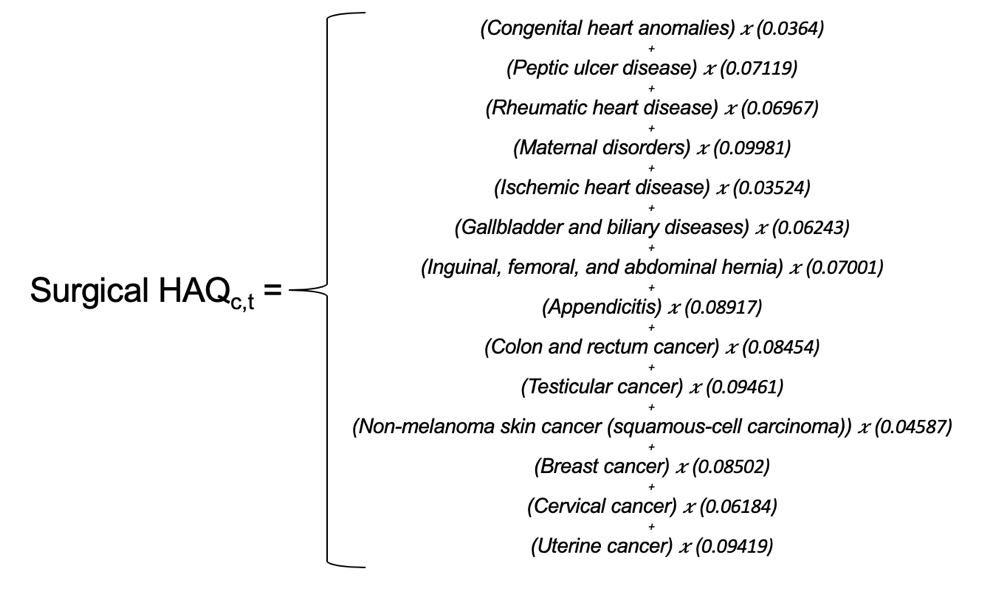


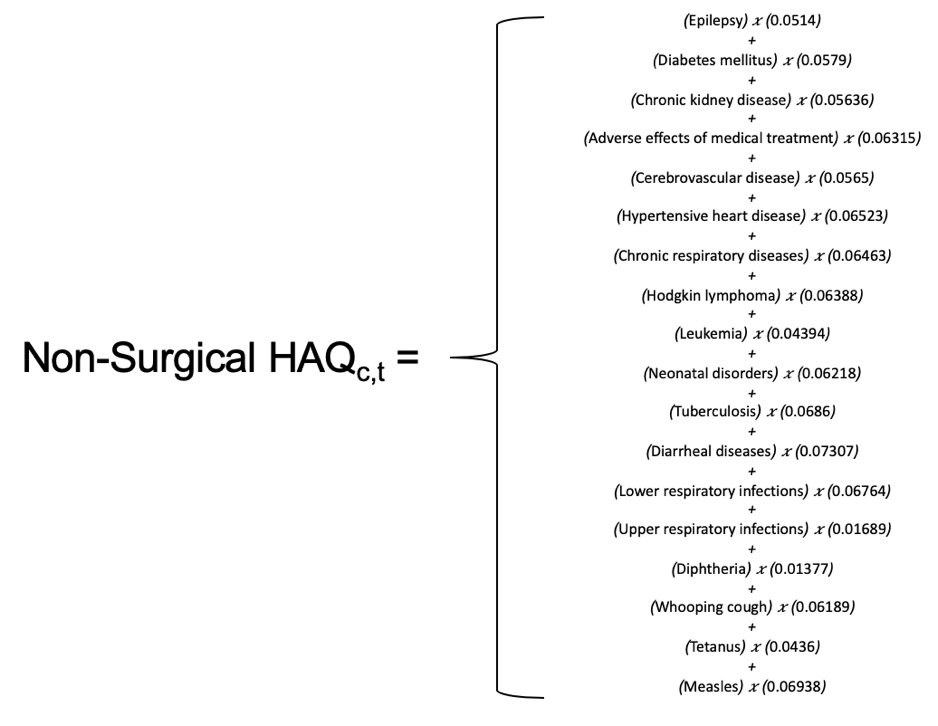

Supplement: S1 Appendix — (DOCX) [file pone.0241669.s008.docx]
